# Enabling equitable and inclusive travel experiences for dysphagia patients: a call to action for the aviation industry

**DOI:** 10.3389/fresc.2026.1623447

**Published:** 2026-04-01

**Authors:** Anthony Cino, Unnathi Annapurna Shashikumar, Kelly Troxell, Mark A. Seeley, Dhruv R. Seshadri

**Affiliations:** 1 Department of Bioengineering, Lehigh University, Bethlehem, PA, United States; 2 Good Shepherd Rehabilitation, Allentown, PA, United States; 3 Orthopedic Research Institute, Geisinger Health System, Danville, PA, United States

**Keywords:** air travel, assistive transportation, dysphagia, equitable healthcare, neuromuscular disease

## Abstract

Dysphagia, or difficulty in swallowing, can be the result of several etiologies such as stroke, neuromuscular disorders, head and neck injuries, or extrinsic compression of the esophagus. Patients with dysphagia are at severe risk of sequelae, such as aspiration and malnutrition, which present a severe risk of mortality. There is a significant need of implementing dysphagia -appropriate menu in commercial airlines. Among the general US population, there is a prevalent increase in dysphagia, which creates a substantial accessibility gap in commercial aviation food service. We reviewed publicly available in-flight menu options from the three largest U.S. carriers (American, United, and Delta) and classified items using the International Dysphagia Diet Standardization Initiative (IDDSI) framework. Across economy-accessible menu items identified online, 56 (American), 67 (United), and 51 (Delta) items were classified to be concentrated in thin liquids (IDDSI 0-1) and regular/easy-to-chew foods (IDDSI 6-7), with no clearly identifiable mildly/moderately thick liquid options (IDDSI 2-3) based on posted descriptions.

## Introduction

Dysphagia, or difficulty swallowing, is a life-threatening condition that greatly impacts the daily health and quality of life of affected individuals. It can be the result of many different etiologies consisting of different types of head and neck injuries, neurological disorders, behavioral disorders, or anatomical anomalies. Dysphagia can result from various conditions that impair swallowing mechanisms, such as swallowing of caustic agents (1), and cervical spinal cord injuries (cSCI) (2). Additionally, these injuries can often originate from intraoperative trauma that result in either nerve damage or compression of the bolus pathway. Moreover, neurogenic dysphagia can be present amongst post-stroke patients (3), patients with neurodegenerative disorders, such as Parkinson's disease (4), and patients with neuromuscular disorders (5), such as multiple sclerosis (6), Duchenne muscular dystrophy (7) and Pompe's disease (8). Furthermore, neurodevelopmental conditions including autism Down syndrome (9) can result dysphagia in these patient populations. Other causes of dysphagia result from various anatomical anomalies which include esophageal tumors (10), esophageal strictures, and cervical spine spondyloses (11). In particular, dysphagia can also present in healthy aging adults, due to their increased susceptibility to several of the aforementioned etiologies, in addition to natural age-relate risk factors, such as decreased motor coordination, decreased tongue strength, and decreased salivary secretions (12, 13). Patients with dysphagia are at severe risk of sequelae that present a high risk of mortality. Dysphagia patients have a higher risk of aspirating sterile gastric contents or food remnants that can lead to aspiration pneumonia, a life-threatening pulmonary infection (14). Furthermore, due to dysphagia patients often experiencing impaired deglutition motility, making it difficult for them to consume their necessary daily nutritional requirements obtained through eating and drinking, these patients may be at further risk for malnutrition and dehydration. This difficulty in ingesting food and beverages can have an extended impact on a patients’ overall wellbeing by disrupting vital metabolic processes and immune system responses (15).

In 2013, 2.7 million US adult hospitalized patients (ages 45–90) were reported to have had a dysphagia diagnosis. Notably, this total does not consider the remaining general population who may have only been diagnosed by outpatient facilities, or who refused to seek out clinical care (16). Hong et al. reported that in 2012 9.44 million adults (4.02% of the general US population) reported having dysphagia, with the estimated prevalence increasing to approximately 15.10 million in 2022 (5.91% of the general US population) (17). Hence, a substantial market size exists for the introduction of accommodating technologies and programs that can ease the quality-of-life burdens that accompany dysphagia. Among these, there exists a significant unmet need in providing suitable dietary options for patients with dysphagia during aviation travel. Commercial aviation presents a unique challenge for individuals with dysphagia due to constrained food preparation environments, standardized catering practices, and limited opportunities for individualized meal modification once onboard. Unlike hospitals or long-term care settings, airline food services are not designed around texture-modified diets, creating a potential mismatch between passenger medical needs and available in-flight options.

**Figure 1:**
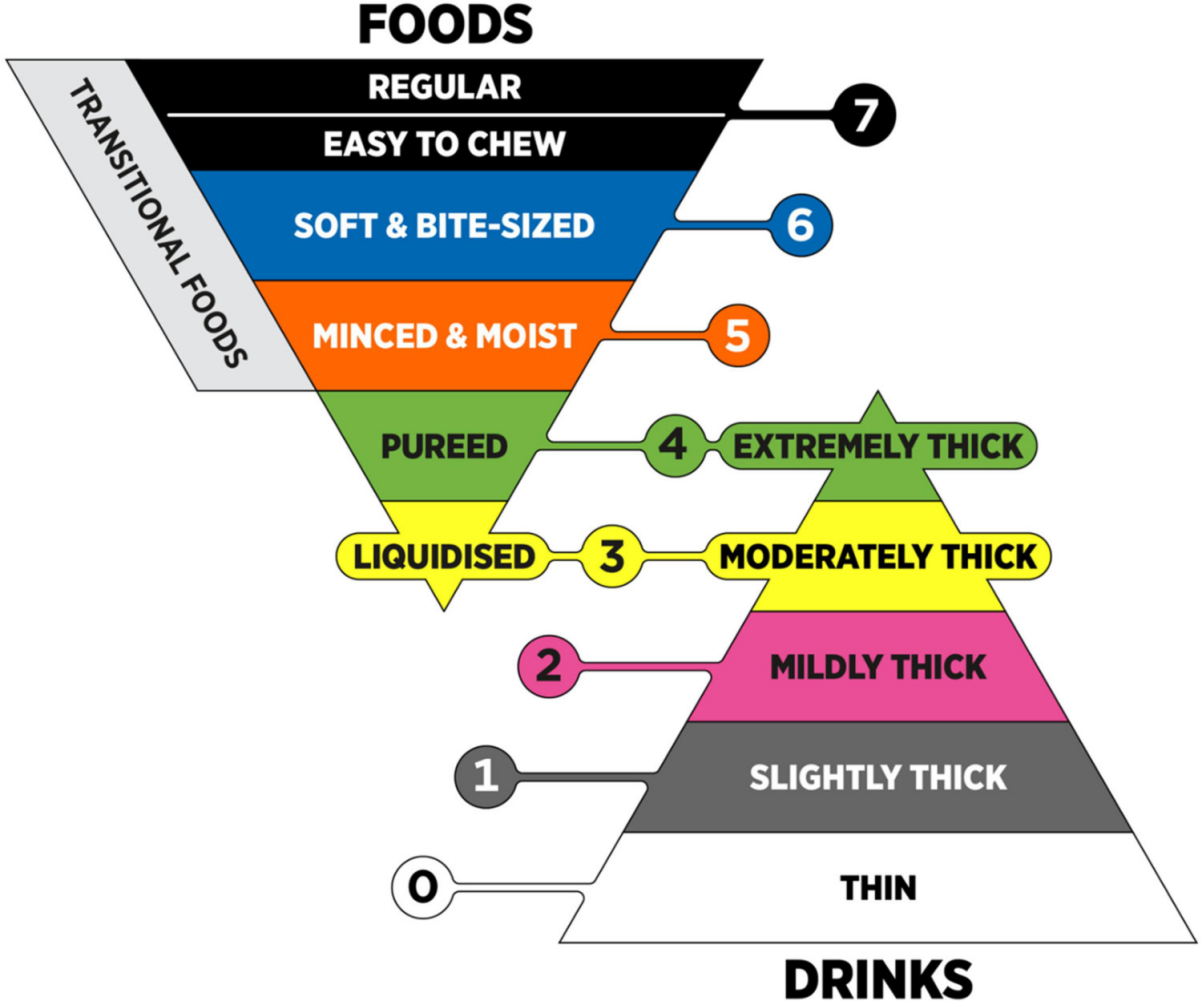
IDDSI framework. Image reproduced with permission from The International Dysphagia Diet Standardization Initiative 2019 @ https://iddsi.org/. Licensed under the Creative Commons Attribution Sharealike 4.0 License https://creativecommons.org/licenses/by-sa/4.0/legalcode (18).

The ideal approach for accommodating individuals with dysphagia during aviation travel should include a standardized system that allows patients to effortlessly alert a flight attendant of their condition, such as by means of a tag or bracelet specifying their individual food and beverage options. This would ensure that these patients can be arranged to receive a wide range of menu options that meet their specific needs either onboard or through prior notifications during travel booking or check-in. The following work will investigate the current menu options that are presently available within the major three carriers in the United States, American, United, and Delta. The food and beverage options that will be discussed from these airlines will be categorized using the International Dysphagia Diet Standardization Initiative (IDDSI) framework (Figure 1) (18). This framework provides a universal classification of different ingestible consistencies, aiming to minimize the risks of choking and aspiration in individualized patients that each have different levels of dysphagia.

## Methods

A descriptive review was conducted of publicly available in-flight menu options from the three largest U.S. commercial airlines: American Airlines, United Airlines, and Delta Air Lines. Menu identification and IDDSI classification were performed by a single reviewer due to resource constraints.

Airline menus were sourced directly from the airlines’ official websites to reflect information available to passengers at the time of review. Because menu formats varied by airline and seating class, each airline's publicly available menus were reviewed individually. Menu options across all seating classes were considered, including snack menus and specialty diet menus such as vegetarian, vegan, gluten-free, and Kosher.

Menu item descriptions were classified using the International Dysphagia Diet Standardization Initiative (IDDSI) framework, which provides standardized definitions for food textures and liquid consistencies used in dysphagia management. Drink items were classified using IDDSI Levels 0–4, while food items were classified using IDDSI Levels 3–7, consistent with IDDSI guidance. Classification was based solely on publicly available menu descriptions, without direct testing or preparation of food items.

When menu descriptions lacked sufficient detail to clearly determine texture or consistency, items were classified conservatively based on the most typical form in which the item is served or were noted as uncertain when classification could not be reasonably inferred. The reviewer recorded the total number of menu items assigned to each IDDSI level for each airline. Descriptive statistics, including counts and proportions of items per IDDSI level, were used to summarize menu distributions across airlines. No inferential statistical testing was performed due to the descriptive nature of the study.

## Results

When identifying the criteria for how accommodating a given menu is for dysphagic patients, a few factors are important to consider. First, there is no clinical evidence to suggest that menu items at one level are objectively best for dysphagic patients. Often dysphagic diets need to be specifically tailored to patients by speech-language pathologists and dieticians. Generally, however, these food and beverage items will typically range from Levels 2–7 in order to avoid highly viscous liquids that may present severe risk for aspiration. For the purposes of this study, the three major American airlines will be evaluated on whether they offer a well-distributed variety of menu options across each of the IDDSI Levels 2–7, ensuring an equal accessibility and choice for all individuals with different dietary needs.

It is important to note that the menu options for each of the following American airlines are subject to frequent changes. Furthermore, these airlines frequently offer specialized meal options that are exclusive to premium membership holders. The following menu listings focus on food and beverage items that are publicly available on the airlines’ official websites and can be reasonably accessed by economy passengers booking a flight with one of the respective airlines.

## Discussion

The three major U.S. airlines evaluated in this study offer diverse menu options intended to enhance passenger comfort and experience during air travel. Across all carriers, a wide range of beverages, including water, coffee, juice, soda, beer, and wine, are consistently available and are categorized as IDDSI Level 0 (thin liquids) (18). In addition, each airline provides special meal accommodations such as vegetarian, vegan, gluten-free, and Kosher options, with some carriers also offering diabetic, low-fat, or lactose-free meals. While these accommodations address certain dietary preferences and restrictions, they do not specifically account for texture and consistency modifications required by many individuals with dysphagia (18).

**Figure 2:**
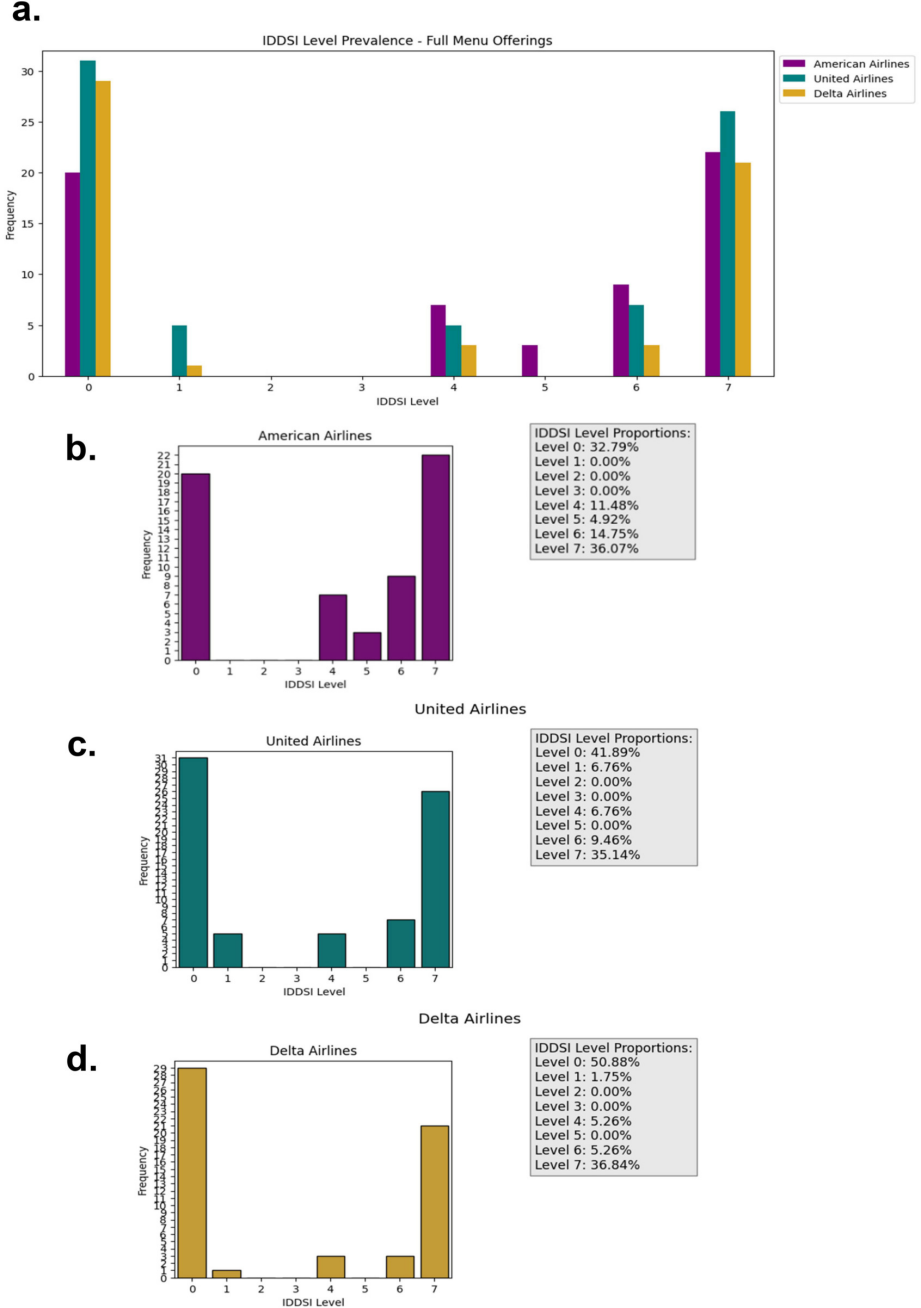
Prevalence and proportion of IDDSI levels for each of the full menu options of the three Major American airlines **(a)** all three **(b)** American airlines **(c)** United airlines **(d)** Delta airlines.

**Figure 3:**
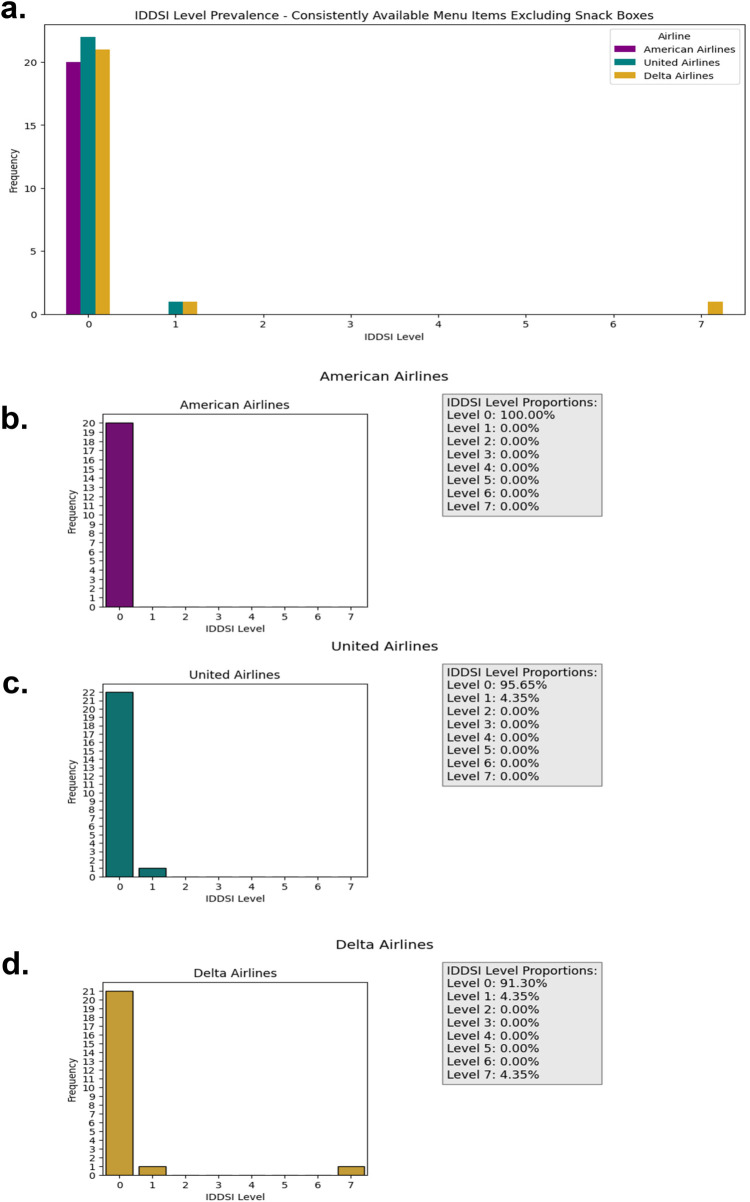
Prevalence and proportion of IDDSI levels for each of menu options of the three Major American airlines excluding snack Box items, restricted items for specific times of the Day, and restricted items based on distance and/or destination **(a)** all three airlines **(b)** American airlines **(c)** United airlines **(d)** Delta airlines.

When evaluating menu items across IDDSI Levels 2–7, results from publicly available airline menus reveal a substantial lack of options suitable for a broad range of dysphagia severities. The full listing of identified menu items and their IDDSI classifications is presented in Table 1, while the prevalence of items across IDDSI levels is summarized in Figures 2, 3. Figure 2 depicts the overall distribution of all identified menu options by IDDSI level, whereas Figure 3 excludes items restricted to snack boxes, specific times of day, or flight distance and destination requirements. This distinction was included to assess how temporal and logistical constraints further limit accessibility for passengers with dysphagia. Such restrictions may exacerbate feelings of exclusion among dysphagic travelers, particularly during long-duration flights where alternative food options are limited or unavailable (14, 15).

**Table 1:** Full listing of menu options for American airlines, United airlines, and Delta airlines categorized based on the IDDSI scale.

American airlines (23)			United airlines (24)			Delta airlines (25)		
Food/beverage	IDDSI level	Comments	Food/beverage	IDDSI Level	Comments	Food/beverage	IDDSI Level	Comments
Coke	0		Coca-Cola®	0		Coca-Cola®	0	
Diet coke	0		Coca-Cola® Zero Sugar	0			0	
Coke zero	0		Diet Coke®	0		Diet Coke®	0	
Dr. Pepper	0		Sprite®	0			0	
Diet Dr. Pepper	0		Seagram's® Ginger Ale	0		Coca-Cola® Zero Sugar	0	
Sprite	0		Seagram's® Seltzer Water	0		Sprite®	0	
Diet sprite	0		Seagram's® Tonic Water	0		Seagram's® Ginger Ale	0	
Canada dry club soda	0		AHA® Sparkling Water	0		Orange Juice	0	
Canada dry tonic water	0		Minute Maid®	0		Cranberry Juice	0	
Canada dry ginger ale	0		Mott's®	0		Fever-Tree Tonic Water	0	
FreshBrew™ coffeehouse Roast	0		Mr & Mrs T® Bloody Mary Mix	0		Fever-Tree Club Soda	0	
FreshBrew™ decaffeinated coffeehouse roast	0		illy® Dark Roast coffee	0		Mr & Mrs T Bloody Mary Mix	0	
Bigelow tea	0		illy® Cold Brew	0		Coffee/Tea[Half & Half, Oat Milk, and Assorted Sweeteners available]	0	
Mott's tomato juice	0		Twinings® Hot Tea	0		Starbucks® Coffee	0	
Mr. & Mrs. T Bloody Mary mix	0		DASANI® Bottled Water	0		Thrive Farmers™ Hot Tea	0	
Minute maid apple juice	0		Michelob ULTRA®	0		Purified Water	0	
Minute maid cranberry cocktail	0		Stella Artois®	0		Bacardí® Rum	0	
Minute maid orange juice	0		White Claw® Hard Seltzer—Black Cherry	0		Buffalo Trace Distillery® Bourbon Cream	1	
Bottled water	0		Kona Big Wave® Golden Ale®	0		Du Nord Foundation Vodka	0	
LaCroix lime sparkling Water	0		Bell's® Two Hearted® IPA	0		Lunazul® Blanco Tequila	0	
Biscoff cookies	7		Just Enough Cabernet Sauvignon	0	*Canned wine available in Domestic Economy. Complimentary red and white wine is available on select long-haul international flights and in domestic premium cabins.	Jack Daniel's® Tennessee Whiskey	0	
Pretzels	7		Just Enough Chardonnay	0	*Canned wine available in Domestic Economy. Complimentary red and white wine is available on select long-haul international flights and in domestic premium cabins.	Woodford Reserve® Kentucky Straight Bourbon Whiskey	0	
Select harvest sea salt roasted almonds	7		Just Enough Rosé	0	*Canned wine available in Domestic Economy. Complimentary red and white wine is available on select long-haul international flights and in domestic premium cabins.	Dewar's® Blended Scotch Whisky	0	
Doritos minis cool ranch	7		Maker Brut Bubbles	0	*Canned wine available in Domestic Economy. Complimentary red and white wine is available on select long-haul international flights and in domestic premium cabins.	Bombay Sapphire® Dry Gin	0	
Fruit and cheese plate			Bacardi® Silver Rum	0		Miller Lite®	0	
*Fruit and cheese	6		Jack Daniel's® Whiskey	0		SweetWater® 420 Extra Pale Ale	0	
*Crackers and chocolate	7		Bombay Sapphire® Dry Gin	0		New Belgium® Voodoo Ranger® IPA	0	
healthy harvest bowls			Bailey's® Irish Cream	1		Une Femme ‘The Betty’ Sparkling Brut	0	
*Bowl	7		Buffalo Trace® Kentucky Straight Bourbon Whiskey	0		Une Femme California Red Blend	0	
*Side of hummus	4		Glenfarclas® Single Malt Scotch	0		Une Femme California Chardonnay	0	
Golden chicken plates			Tito's® Handmade Vodka	0		Lotus Biscoff® Cookies	7	
*Chicken	7		Corazón® Blanco Tequila	0		88 Acres Dark Chocolate Sea Salt Seed + Oat Bar	7	
*Green beans	7		Crafthouse Cocktails Pineapple Daiquiri	1		30 K Snack Mix	7	
*Roasted tomato chutney	4		Crafthouse Cocktails Espresso Martini	1		SunChips® Garden Salsa® Flavored Whole Grain Snacks	7	
*Salted smashed potatoes	4		Crafthouse Cocktails Mai Tai	1		BISTRO SNACK BOX		*Available on flights over 900 miles.
Maple strawberry chia overnight oats	4	*Exclusive to Admirals Club in Austin, TX; (AUS)	Crafthouse Cocktails Moonlighter Vodka Spritz	1		*Potato Chips	7	
Roasted jalapeno hummus	4	*Exclusive to Admirals Club in Austin, TX; (AUS)	Undercover Quinoa Crisp	7	*Available to buy all day on select flights over 500 miles within the U.S., Canada, Latin America, and the Caribbean. Free snacks are available for United Economy® travelers on flights over 300 miles.	*Crackers	7	
Banana caramel bread pudding	5	*Exclusive to Admirals Club in Boston, MA; (BOS)	Savory Snack Mix	7	*Available to buy all day on select flights over 500 miles within the U.S., Canada, Latin America, and the Caribbean. Free snacks are available for United Economy® travelers on flights over 300 miles.	*Cheese Spread	4	
Honey miso shrimp, vegetable yakisoba, crispy onions, hoisin sauce and scallions	7	*Exclusive to Admirals Club in Boston, MA; (BOS)	Daelmans Stroopwafel	7	*Available to buy all day on select flights over 500 miles within the U.S., Canada, Latin America, and the Caribbean. Free snacks are available for United Economy® travelers on flights over 300 miles.	*Beef Snack Stick	7	
Mixed berry French toast	6	*Exclusive to Admirals Club in Charlotte, NC; (CLT)	Pringles®	7	*Available to buy all day on select flights over 500 miles within the U.S., Canada, Latin America, and the Caribbean. Free snacks are available for United Economy® travelers on flights over 300 miles.	*Almonds	7	
Sweet and sour pork with pineapple, brown rice and quinoa, toasted sesame seeds, scallions and chili crisp	7	*Exclusive to Admirals Club in Charlotte, NC; (CLT)	Unna Bakery Brown Butter Cookies	7	*Available to buy all day on select flights over 500 miles within the U.S., Canada, Latin America, and the Caribbean. Free snacks are available for United Economy® travelers on flights over 300 miles.	*Chocolate Sandwich Cookie	7	
Mediterranean breakfast egg scramble	6	*Exclusive to Admirals Club in Chicago, IL; (ORD)	CB&J Mix	7	*Available to buy all day on select flights over 500 miles within the U.S., Canada, Latin America, and the Caribbean. Free snacks are available for United Economy® travelers on flights over 300 miles.	*Gummy Bears	7	
Green goddess cabbage feta salad	7	*Exclusive to Admirals Club in Chicago, IL; (ORD)	Albanese True Fruit Gummi Bears	7	*Available to buy all day on select flights over 500 miles within the U.S., Canada, Latin America, and the Caribbean. Free snacks are available for United Economy® travelers on flights over 300 miles.	MARKET SNACK BOX		*Available on flights over 900 miles.
Cheddar omelet fold with chipotle chimichurri sauce	6	*Exclusive to Admirals Club in Dallas-Fort Worth, TX; (DFW)	Tapas Snackbox		*Available to buy all day on select flights over 500 miles within the U.S., Canada, Latin America, and the Caribbean. Free snacks are available for United Economy® travelers on flights over 300 miles.	*Pita Chips	7	
Argentina beef empanada with roasted red pepper chimichurri	7	*Exclusive to Admirals Club in Dallas-Fort Worth, TX; (DFW)	*Traditional Hummus	4		*Hummus	4	
Maple strawberry chia overnight oats	4	*Exclusive to Admirals Club in Denver, CO; (DEN)	*Pita Chips	7		*Bruschetta Spread	4	
Honey mustard pork loin with spring onion and sage	7	*Exclusive to Admirals Club in Denver, CO; (DEN)	*Bruschetta Dip	4		*Olives	6	
Roasted tomato feta breakfast wrap with salsa verde	6	*Exclusive to Admirals Club in Los Angeles, CA; (LAX)	*Rosemary Cracker	7		*Dried Fruit	7	
Orange peel tempura chicken with roasted red pepper and onion	7	*Exclusive to Admirals Club in Los Angeles, CA; (LAX)	*Poshi® green olives	6		*Almonds	7	
Scrambled eggs with parmesan and tomato	5	*Exclusive to Admirals Club in Miami, FL; (MIA)	*Cracked Black Pepper Almonds	7		*Ginger Chew	7	
Build-your-own tacos with spicy chicken, roasted veggies, soft tortillas and shredded cheddar	7	*Exclusive to Admirals Club in Miami, FL; (MIA)	Takeoff Snackbox		*Available to buy all day on select flights over 500 miles within the U.S., Canada, Latin America, and the Caribbean. Free snacks are available for United Economy® travelers on flights over 300 miles.	CHICKEN SALAD SANDWICH PLATE		*Available on select flights over 1,500 miles departing between 5:00 a.m. and 8:59 p.m.
Southwest salsa verde breakfast wrap	7	*Exclusive to Admirals Club in Nashville, TN; (BNA)	*Beef Salami	7		*Herb Chicken Salad Sandwich	7	
Edamame shrimp teriyaki with carrots and water chestnuts	7	*Exclusive to Admirals Club in Nashville, TN; (BNA)	*Garlic and Herb Crostini	7		*Apple Slices	7	
Build-your-own breakfast slider with egg patty and cheddar cheese	7	*Exclusive to Admirals Club in New York, NY; (LGA)	*Gouda Cheese Spread	4		*Dark Chocolate Square	7	
Fig and balsamic chicken with caponata brown rice with mozzarella, walnuts, lemon wedges, garlic chips and crumbled blue cheese	7	*Exclusive to Admirals Club in New York, NY; (LGA)	*Sourdough Flatbread Crackers	7		FRUIT & CHEESE PLATE		*Available on select flights over 1,500 miles departing between 5:00 a.m. and 8:59 p.m.
Raspberry chia pudding	4	*Exclusive to Admirals Club in Philadelphia, PA; (PHL)	*Divina Fig Spread	4		Gouda and Cheddar Cheese Slices	6	
Chickpea edamame salad with mint and lemon	7	*Exclusive to Admirals Club in Philadelphia, PA; (PHL)	*Karma Olive Oil Cashews	7		Olive Oil & Sea Salt Flatbread Crackers	7	
Turmeric blueberry quinoa salad with cashews	7	*Exclusive to Admirals Club in Phoenix, AZ; (PHX)	*Hazelnut Wafer	7		Whole Natural Almonds	7	
Southwest bean salad	7	*Exclusive to Admirals Club in Phoenix, AZ; (PHX)	Recline Snackbox		*Available to buy all day on select flights over 500 miles within the U.S., Canada, Latin America, and the Caribbean. Free snacks are available for United Economy® travelers on flights over 300 miles.	Dried Apricots	7	
Biscuit and chicken sausage gravy with cheesy scrambled eggs	5	*Exclusive to Domestic Routes	*Community Snacks Kettle Cooked Potato Chips	7		Red Grapes	6	
White cheddar and Monterey Jack omelet with chicken sausage and seasoned wedge potatoes	6	*Exclusive to Domestic Routes	*Vermont Beef Stick	7		Dark Chocolate Square	7	
Smothered veggie and cheese frittata with tomato wedges and everything seasoned potatoes	6	*Exclusive to Domestic Routes	*Pork Stick	7				
Swiss and veggie omelet with Italian chicken sausage and seasoned wedge potatoes	6	*Exclusive to Domestic Routes	*Albanese® True to Fruit gummi bears	7				
A three-cheese omelet with seasoned wedge potatoes	6	*Exclusive to Domestic Routes	*OREO® cookies	7				
			*Chocolate Cranberry Trail Mix	7				
			Everything Bagel Dots with Plain Cream Cheese	6	*Available for purchase on flights over 1,190 miles (U.S. exclusive)			
			Steak, Egg, Cheese Burrito	6	*Available for purchase on flights over 1,190 miles (U.S. exclusive)			
			Cinnamon Rolls with Cream Cheese Frosting	6	*Available for purchase on flights over 1,190 miles (U.S. exclusive)			
			United Airlines Signature Item—The Honey G Cheeseburger by Jeff Mauro	7	*Available for purchase on flights over 1,190 miles (U.S. exclusive) *Served onboard flights from 10:00 AM to 8:59 PM.			
			Cheese Lasagna with Cauliflower Bolognese Sauce	6	*Available for purchase on flights over 1,190 miles (U.S. exclusive) *Served onboard flights from 10:00 AM to 8:59 PM.			
			Fresh Cheese & Fruit Tray	6	*Available for purchase on flights over 1,190 miles (U.S. exclusive) *Served onboard flights from 10:00 AM to 8:59 PM.			
			Maple Breakfast Sandwich	6	*Served onboard flights from 5:00 AM to 9:59 AM for U.S. and Latin America/Caribbean			
			Hummus Platter	4	*Available for purchase on flights over 1,190 miles (U.S. exclusive) *Served onboard flights from 10:00 AM to 8:59 PM.			
			Forty Creek Barbecue Burger	7	*Available for purchase on flights over 1,190 miles (U.S. exclusive) *Served onboard flights from 10:00 AM to 8:59 PM.			
			Sun-dried Tomato Chicken Pesto Wrap	7	*Available for purchase on flights over 1,190 miles (U.S. exclusive) *Served onboard flights from 10:00 AM to 8:59 PM.			
			Turkey and Mozzarella Sandwich	7	*Available on select flights over 1,190 miles from Latin America and the Caribbean to the U.S., excluding long-haul routes with free meal service in Economy class *Served onboard flights from 10:00 AM to 8:59 PM.			
			BBQ Chicken Sandwich	7	*Available on select flights over 1,190 miles from Latin America and the Caribbean to the U.S., excluding long-haul routes with free meal service in Economy class *Served onboard flights from 10:00 AM to 8:59 PM.			

* Provided in the table means - exclusive to airlines/ class.

Across all airlines, menu offerings were heavily concentrated at IDDSI Level 0 (thin liquids) and Level 7 (regular foods). While certain individuals with mild dysphagia may safely tolerate Level 7 foods, this does not adequately address the needs of patients who require texture-modified or thickened consistencies. Individuals with dysphagia vary widely in their swallowing capabilities and nutritional requirements (18), and many may require lower-level options (e.g., Levels 2–6) to eat safely. The limited availability of such options highlights a significant accessibility gap in current airline food service practices and raises concerns regarding passenger safety, nutritional adequacy, and overall inclusivity (14, 15).

No inferential statistical testing was performed, as this study was designed as a descriptive review of publicly available menu information. The intent was to characterize existing menu offerings and identify gaps relative to a standardized dysphagia diet framework, rather than to draw statistical inferences about differences between airlines. Nevertheless, the observed absence or scarcity of menu items at several IDDSI levels across all three carriers consistently points to a systemic lack of dysphagia-appropriate accommodations in commercial aviation.

The SWOT analysis included in this study was intended as a qualitative, industry-oriented framework to contextualize potential implementation pathways for dysphagia accommodations. While SWOT analysis is useful for identifying operational strengths, limitations, opportunities, and threats, it inherently involves subjective interpretation and does not provide quantitative decision-making rigor. Future research could build upon the present findings by incorporating more structured analytical frameworks, such as gap analyses of IDDSI-aligned menu availability, benchmarking against healthcare or other transportation catering systems, multi-criteria decision analysis to evaluate intervention feasibility, or cost-effectiveness and policy gap analyses to assess regulatory and economic considerations.

The discussion of wearable neuromuscular electrical stimulation (NMES) technology is included as a forward-looking perspective rather than as a component of the present menu analysis. Wearable NMES devices have demonstrated potential in dysphagia rehabilitation (22) and may, in the future, serve as adjunctive tools to support safer swallowing in non-clinical environments. However, such technologies should be viewed as complementary rather than replacements for appropriate dietary accommodations. Ensuring access to safe and suitable food and beverage options remains a foundational requirement for inclusive travel experiences among individuals with dysphagia.

This study relied on publicly available online menu descriptions, which may not fully capture variations in food preparation, texture, or moisture content that occur during in-flight service. Menu offerings may also vary by route, aircraft type, and catering provider. Additionally, menu classification was conducted by a single reviewer, which may introduce subjective bias despite the use of a standardized framework (18). Resource constraints prevented the use of multiple reviewers or inter-rater reliability assessment. Finally, the study focused exclusively on U.S. airlines, which may limit generalizability to international carriers with different regulatory environments and catering practices.

Despite these limitations, this work highlights a clear and underexplored accessibility gap in commercial aviation. As air travel continues to serve an increasingly diverse and aging population, addressing the needs of passengers with dysphagia will be essential to promoting safety, dignity, and inclusion. The findings underscore the need for airlines to consider texture-modified dietary accommodations as part of broader accessibility initiatives and provide a foundation for future research and policy development in this area.

## Conclusion

To support safe swallowing for individuals with dysphagia, airlines should consider expanding the availability of food and beverage options spanning IDDSI Levels 2–7. Many individuals with dysphagia are unable to safely consume thin liquids classified as IDDSI Levels 0–1 due to increased aspiration risk (19) and instead require thickened liquids or texture-modified foods tailored to their swallowing needs.

Although passengers may bring their own thickening agents for in-flight use, this option can present practical challenges, particularly in the context of aviation security procedures governing liquids. While medical exceptions exist, reliance on personal accommodations places additional responsibility on passengers and may limit equitable access to safe nutrition during air travel. Providing onboard alternatives could therefore reduce this burden and improve inclusivity.

From an implementation perspective, several potential interventions can be considered across different time horizons. Short-term, lower-barrier strategies include offering commercially available thickening agents upon request or ensuring that at least one thickened beverage or puréed food option is available during meal service. Longer-term strategies may involve modifying existing menu items through puréeing or partnering with commercial suppliers of pre-prepared texture-modified meals designed for dysphagia management. These approaches would require greater coordination with catering services but may offer more comprehensive and consistent accommodations.

The range of feasible options and their respective strengths, limitations, opportunities, and challenges are summarized through a qualitative SWOT analysis (Table 2). Together, these findings underscore the need for airlines to consider dysphagia-related dietary accessibility as part of broader inclusive service initiatives and provide a foundation for future research and policy development in this area.

**Table 2:** SWOT analysis of available solutions for incorporating a dysphagia diet in airline flight menus.

Option	Description	Strengths	Weaknesses	Opportunities	Threats
Purée diet	Diet consisting only of yogurt, bananas, pudding, avocados, and other foods of similar consistency	• Cost-effective • Vitamin-rich • Minimal preparation	• Limited variety • Customers may feel excluded from larger, savory menu options	Diversifying menu options that can be provided to patients with or without dysphagia	Customers may feel more excluded compared to other passengers and may be more inclined to redirect business elsewhere
Mechanically homogenized foods	Menu consisting of current menu options that are homogenized and blended	• Customers have similar access to entire menu	• Additional labor • Time-consuming	Customers may feel the most included compared to other options	Flight attendant burnout/ workforce increase
Thickening powder/gel	Current beverage options with added purchased thickening powder or gel (19)	• Customers can enjoy any beverage of their choosing	• Cost-prohibitive	Increased beverage sales	Potential profit loss due to investment in large quantity thickening powders/gels
Commercial puréed foods	Pre-prepared, purchased food menu options from brands such as Hormel Health labs, Blossom Foods, or Dysphagia Diet among others	• Minimal preparation	• Cost-prohibitive • Single-use servings	Partnerships with nutritionists to promote best pre-prepared food brand	Market competition from other pre-prepared food brands

Furthermore, given that wearable electrode technology is permitted by the TSA for passenger carry-on, patient swallowing during long flights can be significantly improved beyond dietary measures through the use of smart technologies that permit electroceutical therapy while concurrently monitoring swallowing capabilities. For appropriate patients, neuromuscular electrostimulation (NMES) has shown to be more effective than traditional exercise-based swallowing alone (20) while providing similar anatomical advantages, such as neurogenesis and improved skeletal muscle hypertrophy and repair (21). Carnaby-Mann et al. found that in a synthesis of acceptable studies (*n* = 7) analyzing the application of NMES for swallowing therapy, that a mean score improvement of 20% in swallowing performance was detected following NMES treatment (22). Moreover, combining this with a real-time risk stratification model that assesses therapeutic efficacy via wireless telemetry that is compatible in airplane mode and with FAA-compliant battery technology would give the patient a much clearer insight into their current condition and progress. Alongside the implementation of dysphagia-accommodating meals, which is an immediate call-to-action that needs to be addressed, the following serves as a future step in catering to passengers during air travel that requires comprehensive support for swallowing difficulties.

## Data Availability

The original contributions presented in the study are included in the article/Supplementary Material, further inquiries can be directed to the corresponding author.
